# Exploring the Therapeutic Potential of Quadripulse rTMS over the Visual Cortex: A Proof-of-Concept Study in Healthy Volunteers and Chronic Migraine Patients with Medication Overuse Headache

**DOI:** 10.3390/biomedicines12020288

**Published:** 2024-01-26

**Authors:** Alessandro Viganò, Tullia Sasso D’Elia, Simona Liliana Sava, Alfredo Colosimo, Vittorio Di Piero, Delphine Magis, Jean Schoenen

**Affiliations:** 1 Headache Research Unit, Department of Neurology, University of Liège, Citadelle Hospital, 4000 Liège, Belgium; 2 IRCCS San Raffaele Alla Pisana, 00163 Rome, Italy; 3 Headache Clinic of Valdor—ISOSL, 4020 Liège, Belgium; 4 Department of Anatomy, Histology, Forensic Medicine and Orthopedics Sapienza, Sapienza—University of Rome, 00185 Rome, Italy; 5 Subintensive Neurology & Headache Centre, Department of Human Neurosciences, Sapienza—University of Rome, 00185 Rome, Italy; 6 Neurology Department and Pain Clinic (CMTD), CHR East Belgium, 4800 Verviers, Belgium

**Keywords:** chronic migraine, medication overuse headache, quadripulse rTMS, visual evoked potentials, central sensitization, neuromodulation

## Abstract

In chronic migraine with medication overuse (CM-MOH), sensitization of visual cortices is reflected by (i) increased amplitude of stimulus-evoked responses and (ii) habituation deficit during repetitive stimulation. Both abnormalities might be mitigated by inhibitory transcranial neurostimulation. Here, we tested an inhibitory quadripulse repetitive transcranial magnetic stimulation (rTMS-QPI) protocol to decrease durably visual cortex excitability in healthy subjects (HS) and explored its therapeutic potential in CM-MOH patients. Pattern-reversal visual evoked potentials (VEP) were used as biomarkers of effect and recorded before (T1), immediately after (T2), and 3 h after stimulation (T3). In HS, rTMS-QPI durably decreased the VEP 1st block amplitude (*p* < 0.05) and its habituation (*p* < 0.05). These changes were more pronounced for the P1N2 component that was modified already at T2 up to T3, while for N1P1 they were significant only at T3. An excitatory stimulation protocol (rTMS-QPE) tended to have an opposite effect, restricted to P1N2. In 12 CM-MOH patients, during a four-week treatment (2 sessions/week), rTMS-QPI significantly reduced monthly headache days (*p* < 0.01). In patients reversing from CM-MOH to episodic migraine (*n* = 6), VEP habituation significantly improved after treatment (*p* = 0.005). rTMS-QPI durably decreases visual cortex responsivity in healthy subjects. In a proof-of-concept study of CM-MOH patients, rTMS-QPI also has beneficial clinical and electrophysiological effects, but sham-controlled trials are needed.

## 1. Introduction

Chronic Migraine (CM) (ICHD-3 1.3) is the most disabling migraine subtype and frequently complicated by medication overuse headache (MOH) (ICHD-3 8.2), due to the overconsumption of acute migraine drugs [1]. CM-MOH patients are difficult to treat and may become resistant to classical prophylactic treatments [2].

Despite the recent avenue of more efficient prophylactic medications like the monoclonal antibodies blocking CGRP neurotransmission, a significant proportion of CM-MOH patients remain severely disabled, at risk of acute medication overuse relapse, and in need of better management [3,4]. There is thus still room for alternative treatment modalities, including non-invasive brain stimulation methods (NIBS) that have the advantages of avoiding drug-to-drug interactions and being quasi devoid of adverse effects contrary to most preventive drugs (see reviews [5,6,7]).

Among the various NIBS, transcranial magnetic stimulation (TMS) is one of the most reliable to modify the excitability of the underlying cortex [8]. Single TMS pulses (sTMS) disrupt transient brain activity and are able to interrupt cortical spreading depression, the culprit of the migraine aura [9]. By contrast, repetitive TMS (rTMS) modifies cortical excitability for longer time periods in a direction that depends chiefly on the stimulation frequency [10,11]. It is an interesting approach in migraine because it allows to design treatment protocols that target distinct facets of migraine pathophysiology and can thus in principle be adapted to the pathophysiological profile of individual patients [12]. The various migraine subtypes have indeed distinct changes in central sensory information processing identifiable with electrophysiological methods and in part reflecting plastic brain proprieties that can be modulated by NIBS [13,14].

In CM, for instance, the amplitude of cortical evoked potentials is increased while it is decreased for a low number of stimuli in interictal episodic migraine (EM), indicating a high pre-activation level of sensory cortices in CM, but a reduced level in EM [15,16]. CM-MOH patients are also characterized by an increased pre-activation level but, contrary to CM patients, they display a habituation deficit on stimulus repetition [17], which is a hallmark of EM between attacks [15]. The functional abnormalities of the migraine brain predominate in the visual areas [18].

The rTMS effects depend on the stimulation frequency. High frequencies activate the underlying cortex while low frequencies have an inhibitory effect. Since the cortical activation varies between migraine subtypes and over the migraine cycle, the rTMS protocol has to be adapted to the electrophysiological profile and can be applied to the most relevant cortical area for optimal therapeutic efficacy [5,7].

Up to now, rTMS trials in CM-MOH have used activating high stimulation rates (10 or 20 Hz) and targeted either the dorsolateral prefrontal cortex (DLPFC) [19,20,21] or the primary motor cortex [22], yielding conflicting results [20,21,23]. A meta-analysis on the subject was published recently [24]. The visual cortex was stimulated only in EM patients and with sTMS, showing an acute effect on the headache in migraine with aura attacks [25] and suggesting a preventive effect after multiple daily pulses also in migraine without aura [26].

For convenient preventive use in clinical practice, rTMS should preferably exert its effect on the brain for long periods, as the available devices are not transportable and need in-hospital treatment. We have shown that high-frequency occipital rTMS, when applied daily for 5 consecutive days, modifies visual evoked potentials (VEP) for several weeks in healthy subjects, but only for 24 to 48 h in EM patients [27].

Quadripulse rTMS (rTMS-QP) is able to induce long-lasting changes in excitability and metaplasticity of the motor cortex [28] and might thus be a stimulation protocol inducing more durable brain excitability changes for migraine prevention. It consists of trains of 4 magnetic pulses delivered at variable interstimulus intervals (ISI) to activate (ISI < 10 ms) or inhibit (ISI > 30 ms) the underlying cortex. We decided to study the ability of rTMS-QP to modify visual cortex responsivity, indexed by VEP, in healthy subjects and CM-MOH patients and, in a second step, to explore its therapeutic potential in a proof-of-concept trial of these patients.

## 2. Materials and Methods

### 2.1. Participants

We recruited 34 subjects: 22 were healthy subjects (HS, mean age 28 ± 10 years, 12 F) belonging to the student community or to the hospital staff, 12 were patients suffering from chronic migraine with medication overuse headache (CM-MOH, mean age 45.9 ± 13.7 years; 11 F) diagnosed according to ICHD-3 criteria 1.3 and 8.2 [1]. We excluded HS with a personal history of recurrent headaches or episodic syndromes in childhood that may be associated with migraine (ICHD-3 1.6) [1], and with a family history of migraine in 1st degree relatives. Subjects suffering from psychiatric and neurological/systemic disorders or with contraindications to transcranial magnetic stimulation [29] were also excluded. CM-MOH patients were allowed to take migraine preventive drugs as long as the dosage was stable for the last 3 months and for the whole trial duration. CM-MOH patients overused NSAIDs (*n* = 5), triptans (*n* = 3) or both (*n* = 4) and had failed at least one attempt of drug withdrawal. Females, unless post-menopausal, were recorded during the 1st half of the menstrual cycle. All subjects provided written informed consent. The study was conducted in accordance with the Declaration of Helsinki, approved by the local Ethics Committee, and was part of a larger trial registered at clinicaltrials.gov (ID NCT02122744).

### 2.2. Visual Evoked Potentials (VEP)

Pattern Reversal-VEP recordings were obtained as follows. Subjects sat in a comfortable armchair in front of a TV screen (Nicolet™-Natus Medical Incorporated, Orlando, FL, USA; 24 × 18 cm) at ±90 cm distance in a quiet, light-dimmed room (200 lux) with the left eye patched. They were instructed to focus on a red dot at the center of the screen. Six hundred alternating black/white checkerboard stimulations were delivered uninterruptedly at a reversal frequency of 3.1 Hz (check size 15 mm, contrast 80%). The cortical signal was recorded with two needle-electrodes inserted subcutaneously at Oz (active electrode) and Fz (reference) according to the international 10–20 EEG system. A ground electrode was placed on the dorsum of the right hand. The electrode position was marked using a red pen in order to keep the same site for all recording sessions.

The signal was amplified with a CED 1902 amplifier (Cambridge Electronic Design Ltd., Cambridge, UK; band-pass filter 0.05–4000 Hz; signal gain set to 1000 times). The 600 evoked responses were analyzed off-line and partitioned into 6 sequential blocks of 100 responses. All recordings were visually analyzed by two trained investigators (AV and TSD) and those containing artifacts were rejected. The N1 peak was defined as the most negative point 60–90 ms after the stimulus, P1 as the most positive peak following N1 between 80–130 ms, and N2 as the most negative point between 90–200 ms. We measured N1P1 and P1N2 amplitudes for each block of 100 responses and evaluated VEP amplitude changes over the 6 successive blocks using a linear regression analysis, a negative slope value indicating habituation, and a positive one potentiation. The amplitude of the 1st VEP block was specifically considered as an index of the cortical preactivation level.

### 2.3. Quadripulse Repetitive Transcranial Magnetic Stimulation (QP rTMS)

We used a Magstim Super Rapid magnetic stimulator (Magstim Co., Ltd., Whitland, Dyfer, UK) connected to a figure-of-eight coil with a maximal stimulation output of 3.5 Tesla. The coil was placed in a vertical position (its handle pointing upward) on the inion-nasion line, with its inferior limit 1 cm above the inion. The intensity of the stimulation was set at 80% of the phosphene threshold (PT) or 90% of the resting motor thresholds (RMT) in subjects who did not report phosphenes. PT was defined as the minimal stimulus intensity at which the subject reported phosphenes in at least 5 out of 10 single TMS pulses, RMT as the lowest stimulation intensity producing a recordable motor evoked potential of ≥50 µV amplitude in the 1st dorsal interosseous muscle (FDI) of the right hand in 5 out of 10 trials.

Quadripulse stimulation consists of a core train of 4 pulses delivered at a varying interstimulus interval (ISI) [28]. For the inhibitory stimulation (rTMS-QPI), trains of 4 pulses were delivered at an ISI of 50 ms and repeated with an inter-train interval of 5 s for 30 min (total of 1440 pulses). Since a pure excitatory protocol (rTMS-QPE) cannot be reliably reproduced using a single Magstim Super-rapid TMS device, we used a primer stimulation with an ISI of 50 ms delivered every 5 s for 10 min over V2–V3, followed by a stimulation with an ISI of 30 ms repeated every 5 s for 30 min over V1 to obtain an excitatory effect, as described by Hamada et al. for the motor cortex [30,31].

### 2.4. Study Design

**Figure 1:**
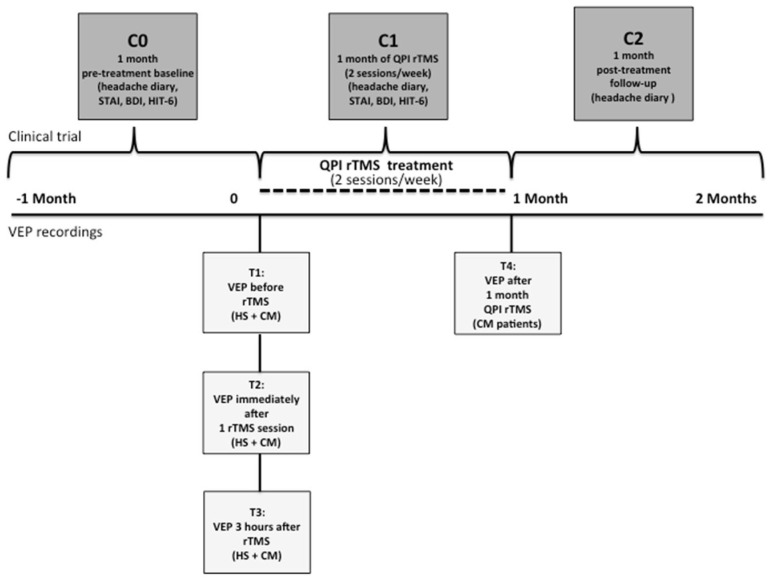
Study design. Flowchart showing the study protocol in HS (VEP recordings) and CM-MOH patients. The time points (T) of VEP recordings are T1 (before rTMS), T2 (immediately after 1 rTMS session), T3 (3 h after the session) in HS and patients, and T4 in patients (after 1 month of twice weekly rTMS-QPI). The time periods for the therapeutic evaluations in CM-MOH patients are C0 (1-month pre-treatment baseline), C1 (during 1 month of twice weekly rTMS-QPI), and C2 (during the post-treatment month).

The study protocol is outlined in Figure 1.

- Electrophysiological study in HS.

HS underwent 3 VEP recordings: before (T1), immediately after rTMS-QP (T2), and 3 h after the stimulation (T3). HS was subdivided into 2 groups of 11 subjects. One group (mean age: 27 ± 10 years, 6 F) received the excitatory protocol (rTMS-QPE), and the other (29 ± 9 years, 7 F) was the inhibitory protocol (rTMS-QPI).

- Proof-of-concept therapeutic and electrophysiological study in CM-MOH patients.

After confirming in HS that the rTMS-QPI protocol effectively and durably changed visual cortex responsivity (see results Section 3.1 below), this stimulation protocol was explored in CM-MOH patients in an open-label, proof-of-concept preventive trial. During the 4 weeks, the patients came twice a week to the laboratory to undergo a 30 min occipital rTMS-QPI session. VEP were recorded at the same time points as in HS (T1, T2, T3), plus an additional recording at the end of rTMS-QPI treatment, i.e., 4 weeks later (T4) (Figure 1). Patients were asked to fill in a headache paper diary to record headache days, headache intensity on a 3-point scale, duration of attacks in hours, associated symptoms like nausea and photo- or phonophobia, and acute medication intake. The number of monthly headache days was the primary outcome measure and was computed from the headache diaries over three different periods: 1 month before the beginning of the trial (baseline, C0), during the 4 weeks of rTMS-QPI (treatment period, C1) and during 4 weeks after the end of the treatment (follow-up, C2) (Figure 1). Before and after treatment all patients completed the HIT-6 and MIDAS questionnaires to evaluate the impact on disability and were evaluated for depression and anxiety using, respectively Beck’s Depression Inventory (BDI) and the State-Trait Anxiety Inventory (STAI-Y2).

Besides the group-level analysis, we searched at the end of the follow-up period for electrophysiological differences between “responders” (*n* = 6), i.e., patients who reverted from chronic to episodic migraine and “non-responders”, i.e., patients who did not (*n* = 6), to identify possible neurophysiological correlates of the therapeutic effect.

### 2.5. Statistical Analyses

We used repeated measures ANOVA (RM-ANOVA) with Tukey’s post-hoc test to analyze VEP habituation slope and 1st block amplitude changes between the T1 (baseline) and the T2–T4 time points as well as clinical changes in CM-MOH patients between C0 and C1–C2. The Mann–Whitney U test was used to estimate differences between groups. Wilcoxon’s rank test was applied to analyze the changes in HIT-6, BDI, and STAI-Y2 scores. Statistics were performed with STATISTICA (version 7 for Windows, StatSoft, Tulsa, OK, USA). Results were considered significant at *p* ≤ 0.05, after proper correction.

## 3. Results

### 3.1. Electrophysiological Effects in Healthy Subjects

In the rTMS-QPI group, one female subject dropped out because she fainted during electrode placement. In the rTMS-QPE group (*n* = 11), the mean phosphene threshold was 65% of maximal output, while in the rTMS-QPI group (*n* = 10) it was 59%. In 2 subjects (one in each group), the visual cortex stimulation did not elicit phosphenes and motor thresholds were therefore used as described in the methods section (rTMS-QPI: MT 50%; rTMS-QPE: MT 63%). At baseline (T1), the 1st block amplitudes and habituation slopes were similar between both rTMS-QPE and rTMS-QPI groups. Results of the electrophysiological study in HS are displayed in Table 1.

In brief, regarding rTMS-QPE, the stimulation induced a consistent modification limited to the P1N2 VEP component and T3 time point. It increased 1st block amplitude of P1N2 at T3 (*p* = 0.03), and at the same time, P1N2 habituation (*p* = 0.02) (Table 1). Conversely, rTMS-QPE had no significant effect on the N1P1 1st block amplitude or on the N1P1 habituation slope at any time point (*p* = 0.9).

**Figure 2:**
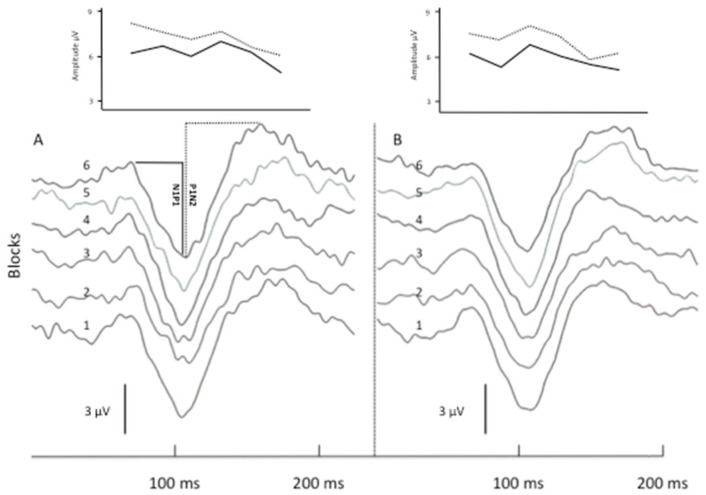
Illustrative VEP recordings before and after rTMS-QPI in a healthy subject. Panels (**A**,**B**) show 6 sequential blocks of 100 averaged VEP responses (numbered 1 to 6) before (**A**) and immediately after (**B**) one session of occipital rTMS-QPI. Above: corresponding amplitude changes of N1P1 (straight line) and P1N2 (dashed line) components.

rTMS-QPI produced a more robust change in VEP responses. Immediately after the stimulation, N1P1 1st block amplitude and N1P1 habituation were unchanged. At T3, however, N1P1 1st block amplitude was reduced and habituation reverted to potentiation (slope shift from −0.04 to 0.04, *p* = 0.03). Compared to T1, P1N2 1st block amplitude was significantly lower both at T2 (*p* = 0.03) and T3 (*p* = 0.02) and consistently rTMS-QPI reverted the pre-stimulation habituation of P1N2 (−0.08) to potentiation both at T2 (+0.01; *p* = 0.04) and T3 (+0.06; *p* = 0.04) (Table 1). Figure 2 shows illustrative VEP recordings (6 sequential blocks of 100 averaged responses) and N1P1 and P1N2 habituation slopes in a healthy subject before and immediately after rTMS-QPI over the visual cortex.

### 3.2. Clinical Effects of rTMS-QPI in CM-MOH Patients

All 12 CM-MOH patients enrolled for the therapeutic proof-of-concept trial completed the 4-week treatment period. Occipital rTMS-QPI was well tolerated and caused no adverse event. Monthly headache days decreased on average from 20 ± 7.2 at C0 (pre-treatment) to 12.3 ± 8.1 at C1 (treatment month) (*p* < 0.01). During C2, i.e., the month following the rTMS-QPI treatment period, the improvement in total headache days remained stable at 12.2 ± 6.7 compared to C0 (*p* = 0.03). Six out of 12 patients reverted from a chronic to an episodic migraine pattern. Overall, the average monthly cumulative time with headache tended to decrease with rTMS-QPI (*p* = 0.07) from a C0 baseline of 240 ± 141 h to 124 ± 150 h at C1 and 156 ± 141 h at C2. The reductions between C0 and C2 in the number of days with severe migraine attacks (grade 3 intensity) (*p* = 0.54) and in acute medication intake (*p* = 0.30) were not significant. While the HIT-6 score tended to improve after treatment (*p* = 0.06), the MIDAS score did not (*p* = 0.71) and STAI (*p* = 0.61) as well as BDI (*p* = 0.50) remained unchanged.

### 3.3. Neurophysiological Effects of rTMS-QPI in CM-MOH Patients: Group Level

**Figure 3:**
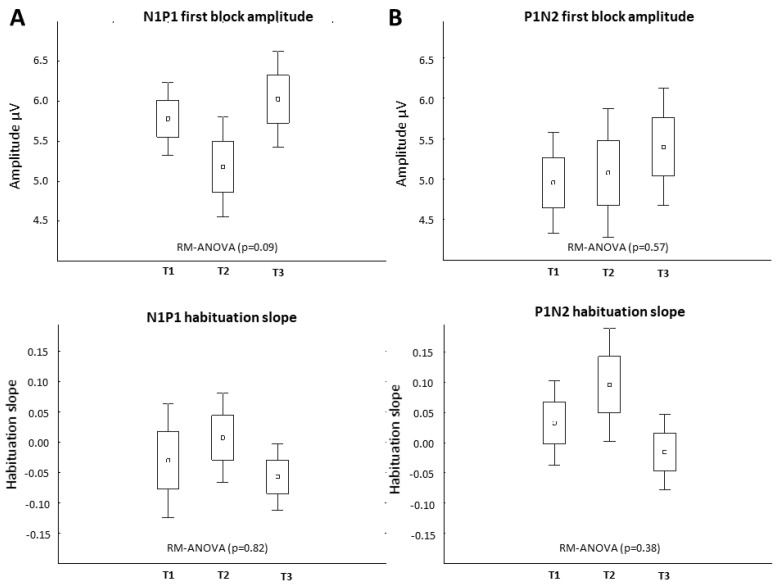
Effect of rTMS-QPI on VEP in CM-MOH patients—group level (*n* = 12). T1: baseline before stimulation, T2: immediately after, and T3: 3 h after one session of occipital rTMS-QPI. Panel (**A**): N1P1 VEP component: 1st block amplitude (**above**) and habituation slope (**below**). Panel (**B**): P1N2 component: 1st block amplitude (**above**) and habituation slope (**below**) (means ± SEM).

Baseline (T1) VEP amplitudes and habituation slopes did not differ between HS and CM-MOH patients, although in the latter VEP habituation slopes were numerically less negative: N1P1: −0.31 ± 0.23 in HS, −0.02 ± 0.2 in CM-MOH (*p* = 0.19); P1N2: −0.10 ± 0.27 in HS, 0.03 ± 0.2 in CM-MOH (*p* = 0.09). No significant VEP change was found in CM-MOH patients at the group level after the 1st rTMS-QPI stimulation at any time point (T1 vs. T2, T3) (*p* = 0.09). There was a numerical decrease in first block amplitude and in habituation at T2, but, unlike in HS, this was not significant and not maintained at T3 (Figure 3). In the total group of CM-MOH patients, there was no significant change in electrophysiological parameters between pre-treatment T1 recordings and those performed at T4 at the end of the rTMS-QPI treatment period.

### 3.4. Neurophysiological Effects of rTMS-QPI in CM-MOH Patients: Responders vs. Non-Responders

“Responders” who reverted from CM to EM, significantly differed from “non-responders”, who remained chronic, in the change of VEP habituation at T4, i.e., at the end of the 1-month rTMS-QPI treatment period (Table 2). In responders, VEP habituation significantly increased while it was unchanged in non-responders, suggesting that clinical benefit was associated with a modification of brain responsivity. This was found for both the N1P1 (*p* = 0.005) and the P1N2 (*p* = 0.05) VEP component. By contrast, there was no significant difference between responders and non-responders in 1st block VEP amplitude, neither for N1P1 (*p* = 0.28) nor for P1N2 (*p* = 0.13).

## 4. Discussion

In the present study, we tested the potential of quadripulse repetitive transcranial magnetic stimulation (rTMS-QP) to modify the responsivity of the visual cortex and hence to be of therapeutic utility in chronic migraine patients with acute medication overuse (CM-MOH) in whom visual processing is abnormal on electrophysiological recordings. We will discuss the effects of excitatory (rTMS-QPE) or inhibitory (rTMS-QPI) quadripulse rTMS on visually evoked potentials (VEP) in healthy subjects (HS), and on VEP and headache in patients.

The effects on the brain of transcranial neurostimulation are only partly understood and vary markedly with the stimulation protocol, which may explain in part discrepancies between published studies [19,20,23]. Recent meta-analyses and systematic reviews of rTMS in migraine have indeed highlighted a large variability of effects that can be summarized as follows: no or small effects compared to transcranial direct current stimulation (tDCS) [32,33], a mild effect weakened by the lack of rigorous adherence to guidelines [34], a different effect depending on the chosen outcome measures [35], a small to moderate beneficial effect of rTMS on motor cortex [36,37], better outcomes in chronic migraine (CM) after rTMS of the dorsolateral prefrontal cortex (DLPFC) but not in all meta-analyses [24,37,38]. The discrepancies between studies are likely multifactorial. Most studies were performed on a small number of patients and underpowered. A variety of different stimulation protocols were used: excitatory or inhibitory stimulations (e.g., high-frequency or low-frequency rTMS or theta-burst) [39], and the timing of the stimulation [24,40]. The rational for targeting the motor cortex or the DLPFC was mainly taken over from trials in chronic pain disorders and depression [11]. Given that responsivity indexed by neurophysiological tests is mainly abnormal in the visual cortex of CM patients and amenable to therapeutic interventions [16,17], one may question whether the motor cortex and DLPFC are the most appropriate targets [41].

For these reasons, we decided to target the visual cortex with a rTMS protocol, quadripulse stimulation, susceptible to producing a robust and lasting change in brain responsivity. Having obtained evidence for such an effect in healthy subjects, we applied the inhibitory protocol (rTMS-QPI) in a proof-of-concept study to treat CM-MOH patients, given that in these patients’ brain responsivity is increased [16].

The rTMS-QP protocol was initially studied over the motor cortex where it induced long-lasting changes in excitability and metaplasticity [28,30]. To the best of our knowledge, the present study is the first to apply rTMS-QP over the visual cortex and show, like for the motor cortex, that it is able in HS to modify VEP in opposite directions depending on the stimulation parameters. The inhibitory paradigm (rTMS-QPI) decreased 1st block VEP amplitude and habituation, confirming that these two VEP parameters are related, as we have previously described in healthy controls and in migraineurs [13,15]. As a corollary, the excitatory protocol with a primer over V2−3 [30] (rTMS-QPE) tended to induce the opposite changes, although with a smaller effect size. The VEP changes induced by rTMS-QP were more pronounced 3 h after than immediately after the neurostimulation session. A similar delay was described for rTMS-QPI over the motor cortex and argues in favor of a prolonged, neuroplastic mode of action [27,30,31].

Our main interest was in the rTMS-QPI protocol because we postulated that it might have a therapeutic effect in CM-MOH patients by mitigating the sensitization of their visual cortex [16,42]. Since rTMS-QPI induced in HS electrophysiological indices of a robust inhibition of the visual cortex, we tested this protocol, as a proof-of-concept, in CM-MOH patients who are notoriously difficult to treat [2]. During a 1-month treatment period of 2 sessions per week, there was a significant clinical improvement with monthly headache days almost halved compared to the pre-treatment month. The clinical benefit outlasted the treatment period and persisted up to 1 month after the end of treatment. Such long-lasting clinical changes were also reported in migraine after rTMS of the primary motor cortex (M1) [23]. Interestingly, half of our patients (*n* = 6) reverted to an episodic migraine pattern (ICHD-3 1.1) [1], while 6 remained chronic. Reversion to episodic migraine is a major objective in chronic migraine management, notably because it allows preventive drugs to become effective again [2].

Contrary to the findings in HS, a single session of occipital rTMS-QPI had no significant effect on VEP parameters in CM-MOH patients, besides inducing a short-lasting numerical decrease of 1st block VEP amplitude. This suggests that in migraineurs repeated sessions of rTMS are needed to induce lasting plastic changes in sensory cortices [27]. After 1 month of twice weekly sessions of rTMS-QPI, there was no significant VEP change globally in CM-MOH patients, but out of 12 patients 6 reverted to an episodic migraine phenotype and were subanalyzed as “responders”. In this subgroup of patients, one month after the rTMS-QPI treatment period, there was a clear-cut increase in VEP habituation, contrary to “non-responders” who tended to have the opposite change. This differs from the VEP changes reported during spontaneous reversion from chronic to episodic migraine, which is generally associated with a shift from normal to deficient habituation, the interictal hallmark of most EM patients [15,16,17]. By the same token, in a cohort of migraine patients of whom one quarter had CM-MOH, 3 sessions of 10 Hz rTMS over M1, but not sham stimulation, improved habituation of somatosensory evoked potentials without significantly changing their amplitude, and this correlated with a decrease in headache intensity [43]. The difference between studies could be due to the fact that the overuse of NSAIDs and/or triptans likely modifies brain excitability and plasticity processes [44,45,46]. As a consequence, the relation between preactivation levels of sensory cortices, i.e., 1st block amplitude of evoked potentials, and habituation could be lost in CM-MOH. Concordantly, an abnormal response of the motor cortex to rTMS at various frequencies was recently reported in CM-MOH as compared to CM or HS [47].

Our study has some obvious limitations. First, we designed a proof-of-concept, pilot study with the aim to explore the effectiveness of a new rTMS protocol and pave the way for larger sham-controlled trials A small number of patients were thus included. Because of its open-label design without a sham stimulation arm, we cannot exclude a placebo effect that is known to be greater with devices than with oral drugs [21,48]. A larger, double-blind, sham-controlled trial is thus mandatory before any firm conclusion can be drawn. Second, the electrophysiological effects of rTMS-QPI in healthy volunteers were studied after a single stimulation session and can thus only partly be compared with those found in CM-MOH patients who underwent multiple sessions. Third, we used the habituation deficit paradigm as a neurophysiological biomarker for treatment effects. Admittedly, the habituation deficit in episodic migraineur patients has not been reproduced in some studies [49]. Several recent studies, however, using high-density EEG and multisensory stimulations have confirmed this abnormality of sensory processing in the migraine brain [50,51]. Moreover, the habituation deficit paradigm is able to capture changes induced by pharmacological and non-pharmacological migraine preventive therapies [17,52,53]. In the future, new neurophysiological instruments, such as high-density EEG responses induced by TMS (TMS-EEG) should contribute to disentangling the underpinnings of hyperresponsivity and abnormal habituation in migraine patients and to identify more precisely the effects of neurostimulation not only for one sensory cortex but for the entire brain [54,55,56,57] as well as regions of interest with abnormal activity [58].

Notwithstanding these limitations, our results with rTMS-QPI are in line with those recently obtained in CM-MOH patients in a randomized, sham-controlled trial of cathodal tDCS over the occipital cortex, another inhibitory non-invasive neurostimulation method [59].

## 5. Conclusions

In conclusion, we tested the hypothesis that a robust and durable neurostimulation protocol inhibiting the visual cortex should have a therapeutic potential in CM-MOH where central sensitization was reported. We chose the protocol of quadripulse rTMS and tested it first in healthy subjects using the visual evoked potential (VEP) as a marker. We found that (i) quadripulse stimulation on the visual cortex had an effect similar to that described originally for the motor cortex [28,30]; (ii) concordant with the habituation theory [10,60,61], an excitatory protocol (rTMS-QPE) increased the first block of VEP averagings and habituation over subsequent blocks, while the inhibitory stimulation (rTMS-QPI) produced opposite changes with a greater effect size. We therefore applied occipital rTMS-QPI in 12 CM-MOH patients as a clinical and electrophysiological proof-of-concept trial. Although rTMS-QPI was unable to induce significant VEP changes, its application twice weekly for 1 month significantly reduced headache burden up to 1 month after the treatment period. In patients reverting from chronic to episodic migraine, this was associated with a significant increase in VEP habituation. These pilot results suggest that a sham-controlled trial of occipital rTMS-QPI is worthwhile in CM-MOH patients using VEP habituation as a marker for central changes.

## Figures and Tables

**Table 1:** VEP changes in healthy subjects induced by excitatory (rTMS-QPE) or inhibitory (rTMS-QPI) quadripulse rTMS over the visual cortex. The last two columns indicate either no significant change (=), a significant increase (🡹), or decrease (🢃) (*p* < 0.05) between pre-rTMS recordings (T1) and recordings performed immediately after (T2) or 3 h after rTMS (T3).

rTMS-QPE						
VEP		T1	T2	T3	T1–T2 Δ	T1–T3 Δ
N1P1	1st Block Amplitude (μV)	6.7 ± 2.44	6.6 ± 2.44	6.6 ± 1.89	=	=
	Habituation Slope	−0.22 ± 0.31	−0.21 ± 0.14	−0.22 ± 0.25	=	=
P1N2	1st Block Amplitude (μV)	5.4 ± 2.38	5.5 ± 1.71	6.3 ± 2.13	=	🡹
	Habituation Slope	−0.13 ± 0.31	−0.14 ± 0.22	−0.27 ± 0.36	=	🢃
**rTMS-QPI**						
**VEP**						
N1P1	1st Block Amplitude (μV)	5.5 ± 2.39	5.3 ± 2.07	5.2 ± 2.34	=	🢃
	Habituation Slope	−0.04 ± 0.16	−0.12 ± 0.24	0.04 ± 0.23	=	🡹
P1N2	1st Block Amplitude (μV)	6.1 ± 2.17	5.2 ± 1.77	5.0 ± 1.99	🢃	🢃
	Habituation Slope	−0.08 ± 0.23	0.01 ± 0.24	0.06 ± 0.19	🡹	🡹

**Table 2:** Relation between therapeutic and electrophysiological effects in CM-MOH patients. Difference between “responders” and “non-responders”: at T4 there is a significant increase in VEP habituation, i.e., a more negative slope (🢃) (*p* < 0.05) in responders, but not in non-responders (=).

rTMS-QPI					
VEP			T1	T4	
1st Block Amplitude	N1P1	Responders	6.39 ± 1.44	7.70 ± 1.75	=
		Non-responders	5.29 ± 1.23	5.23 ± 1.28	=
	P1N2	Responders	5.70 ± 1.75	6.96 ± 0.72	=
		Non-responders	4.36 ± 1.97	4.20 ± 0.66	=
Habituation slope	N1P1	Responders	−0.08 ± 0.36	−0.33 ± 0.26	🢃
		Non-responders	0.01 ± 0.24	0.21 ± 0.36	=
	P1N2	Responders	−0.001 ± 0.19	−0.23 ± 0.25	🢃
		Non-responders	0.06 ± 0.24	0.37 ± 0.46	=

## Data Availability

The data presented in this study are available on reasonable request from the corresponding author. The data are not publicly available due to patients’ privacy.
